# Emergency Colectomies in the Elderly Population—Perioperative Mortality Risk-Factors and Long-Term Outcomes

**DOI:** 10.3390/jcm12072465

**Published:** 2023-03-23

**Authors:** Ilan Kent, Amandeep Ghuman, Luna Sadran, Adi Rov, Guy Lifschitz, Yaron Rudnicki, Ian White, Nitzan Goldberg, Shmuel Avital

**Affiliations:** 1 Department of Surgery, Meir Medical Center, Kfar Saba 4428164, Israel; 2 Sackler Faculty of Medicine, Tel Aviv University, Tel Aviv 6139001, Israel; 3 Department of Surgery, University of British Columbia, Vancouver, BC V6T 1Z4, Canada

**Keywords:** emergent colectomy, elderly, geriatric, mortality, dementia, bedridden

## Abstract

Background: As the population ages emergency surgeries among the elderly population, including colonic resections, is also increasing. Data regarding the short- and long-term outcomes in this population is scarce. Methods: A retrospective study was performed to investigate mortality and mortality risk factors associated with emergent colectomies in older compared to younger patients in a single university affiliated tertiary hospital. Patients with metastatic disease, colectomy due to trauma or index colectomy within 30 days prior to emergent surgery were excluded. Results: Operative outcomes compared among age groups, included 30-day mortality, mortality risk-factors and long-term survival. 613 eligible patients were included in the cohort. Mean age was 69.4 years, 45.1% were female. Patients were divided into four age groups: 18–59, 60–69, 70–79 and ≥80-years. Thirty-day mortality rates were 3.2%, 11%, 29.3% and 37.8%, respectively and 22% for the entire cohort. Risk-factors for perioperative death in the younger group were related to severity of ASA score and WBC count. In groups 60–69, 70–79, main risk-factors were ADL dependency and ASA score. In the ≥80 group, risk-factors affecting perioperative mortality, included ASA score, pre-operative albumin, creatinine, WBC levels, cancer etiology, ADL dependency, and dementia. Long-term survival differed significantly between age groups. Conclusion: Perioperative mortality with emergency colectomy increases with patients’ age. Patients older than eighty-years undergoing urgent colectomies have extremely high mortality rates, leading to a huge burden on medical services. Evaluating risk-factors for mortality and pre-operative discussion with patients and families is important. Screening the elderly population for colonic pathologies can result in early diagnosis potentially leading to elective surgeries with decreased mortality.

## 1. Introduction

The elderly population in the western world is growing rapidly. It is estimated that by 2030, the world is likely to have 1 billion people older than age 65, accounting for 13% of the world population [1]. In the United States, more than 35 million Americans are 65 years or older, which represents more than 12% of the population [2]. This segment of the population is expected to increase very quickly in the near future [3]. General surgery, like many other fields of medicine, is facing a tremendous shift, with accommodations made to treat patients in this age group. It is estimated that more than half of the operations performed in the United States involve patients 65 or older [4]; although they comprise only 15% of the population. Many colonic pathologies requiring surgery, including colorectal cancer, diverticulitis and ischemic colitis, are diseases of aging [5,6,7]. The number of abdominal surgeries performed on elderly patients is expected to increase, including emergent surgeries. Emergent surgery in any population, even more so among the elderly and specifically emergent colorectal surgery, all have higher rates of morbidity and mortality and poorer outcomes [8]. Therefore, it is becoming imperative to investigate modifiable risk-factors, to improve patient outcomes among the elderly. This study evaluated 30-day mortality after emergency colorectal surgery in elderly patients to identify risk-factors for mortality and to evaluate their long-term survival compared to younger patients.

## 2. Methods

### 2.1. Study Design

This retrospective study included all patients ≥18 years of age who underwent an emergent colectomy at a university-affiliated tertiary medical center from 2005 to 2017. Patients were identified from the institutional database. Exclusion criteria were patients who underwent a colostomy as primary surgery, patients with metastatic disease, colonic injury secondary to trauma or a colectomy performed within 30-days secondary to a previous operation. Patients with metastatic disease, including oligometastatic disease, were excluded from this cohort in order to try to investigate the impact of the emergent surgery disregarding the background of potentially terminal disease and its associated significant risk factors such as malnourishment, immunologic deficiency and other related conditions. As we also tried to evaluate long term survival after surgery (up to 12 years) related mainly to the traumatic event of the emergent surgery and not to a metastatic disease.

### 2.2. Study Rationale, Variables, and Outcome Measures

First, we analyzed the primary outcome of 30-day mortality to compare overall survival across the groups, to determine a cut-off value of a meaningful decrease in 30-day survival. A sharp increase in 30-day mortality was observed in the groups older than 60 years. We than aimed to determine risk-factors for 30-day mortality. The cohort was subdivided into four age categories 18–59, 60–69, 70–79 and ≥80-years. Factors assessed for inclusion in the models included gender, body mass index (BMI), etiology for resection, stoma creation, being bed ridden (ADL dependency) diagnosis of dementia, Charlson comorbidity index, blood transfusion, hemoglobin level, white blood cell count (WBC), American Society of Anesthesiologists physical status score (ASA) and creatinine level. Main outcome measures were 30-day mortality and post-operative complications.

### 2.3. Statistical Analysis

Descriptive statistical analysis was performed on patient characteristics, including demographics and operative variables and compared between the various groups using student *t*-test for continuous data and chi squared for categorical data. A *p*-value < 0.05 was considered significant. Univariate logistic regression was performed to identify predictors of 30-day mortality in each age group. Factors with *p*-values < 0.05 were considered significant and included in a multivariable logistic regression analysis. A *p*-value < 0.05 was considered significant in multivariable analysis to determine adjusted odds ratios of risk-factors for 30-day mortality in the four age categories. Statistical analyses were performed using IBM SPSS Statistics for Windows, version 21.0 (IBM Corp., Armonk, NY, USA).

## 3. Results

**Figure 1:**
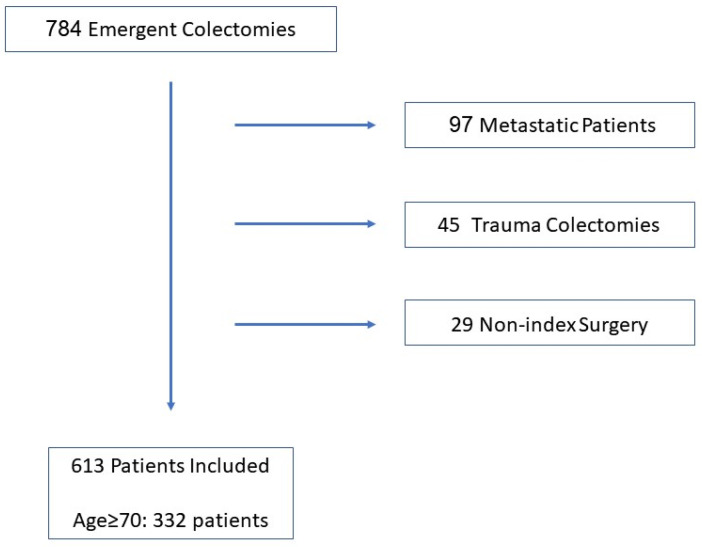
Flow diagram of patient inclusion.

**Figure 2:**
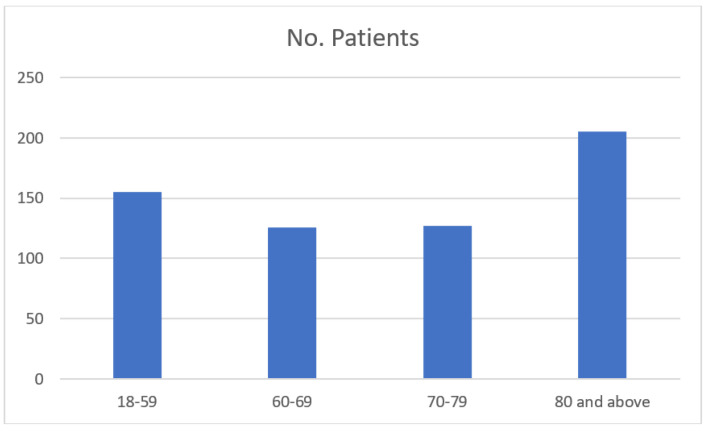
Surgeries in each age group.

**Figure 3:**
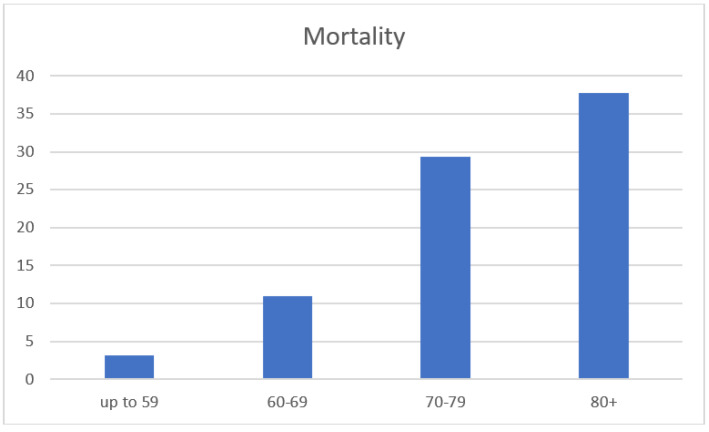
30-day mortality rates in each age group.

**Figure 4:**
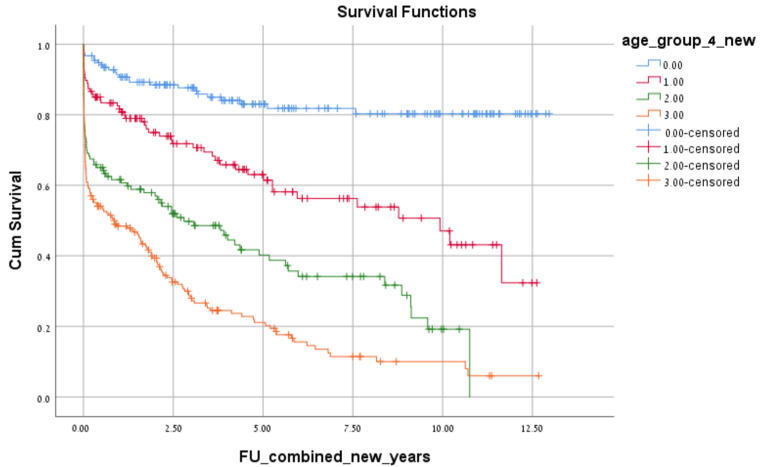
Overall Kaplan-Meier survival curve.

A total of 784 patients who underwent an emergent colectomy from January 2005 to December 2017 were initially identified for chart review. Of these, 613 met the inclusion criteria and were included in the analysis (Figure 1). The cohort included 336 (55%) men and 277 (45%) women. Operation for cancer as an etiology was performed in 408 (66.5%) patients. A diversion with an ostomy was performed in 230 (37.5%) patients. The Average length of stay was 14.4 days (1–107 Days). The median follow-up for the entire cohort was 77 months. The number of emergency operations increased with age, with the largest group of patients ≥80 years (Figure 2). The two most common surgeries performed were right colectomy (*n* = 178) and Hartmann’s procedure (*n* = 178). All procedures performed on the cohort are shown in Table 1. The overall 30-day mortality rate was 22% for the entire cohort. The 30-day mortality in the four age groups was 3.2%, 11%, 29.3% and 37.8%, respectively (Figure 3). In the youngest patient group, perioperative mortality was related to illness status when arriving to surgery, as reflected by ASA scores and WBC counts (Table 2 and Table 3). In the intermediate age groups (69–69, 70–79), there was an incremental increase in mortality rate that was associated with ASA score and ADL dependency (Table 2 and Table 3). The high mortality rate (37.8%) in patients older than 80 years was associated, in a univariate analysis, with ASA score and ADL dependency like in the younger age groups. However, many other risk-factors, including, WBC, creatinine, albumin levels prior to surgery, dementia, cancer etiology and blood transfusion were found to be associated with perioperative mortality in this age group (Table 2). Interestingly, these factors did not reach statistical significance in multivariate analysis. This could be because these factors are much more prevalent in patients older than 80 in general and their combination together leads to a substantial increase in perioperative mortality in this specific age group. Long-term overall survival was also in accordance with the age group (Figure 4). Patients ≥80 years who survived the operation had a 22% mortality rate one year after surgery.

## 4. Discussion

The number of emergency colectomies performed increases as the population ages. As shown in our cohort, these surgeries entail higher mortality rates. This is a growing healthcare concern and a major economic and social burden that will likely intensify as the fraction of the aging population continues to increase.

This study evaluated 30-day mortality rates and risk-factors after emergent colectomies in different age groups. We found an increase in emergent colectomies performed in patients over the age of 80. We also found a substantial, gradual increase in perioperative mortality in patients above 60 years old, reaching to almost 40% among patients 80 or older. These findings are in contrast to younger patient populations where morbidity and mortality rates are similar between elective colorectal resections and emergency colectomies [3,9] and also potentially in contrast to elective colorectal surgery in the elderly were 30 days mortality rates were also lower [10].

The mortality rates in our cohort are slightly higher than those reported in a retrospective review of emergent colorectal surgeries by McGillicuddy and co-workers [11]. Their study revealed a 15% in-hospital mortality in an elderly cohort. However, they defined elderly as patients older than 65. Modini and colleagues reported a 30% mortality rate among 93 patients over 80 years old [12]. This is comparable to the rates in our cohort. In addition, in a review of 1245 patients undergoing colorectal resection, Klima et al. reported a 1.32 times greater likelihood of mortality for each 10 year age increase [13].

The observed high mortality rates after emergency surgery in elderly patients may be due to pre-existing comorbidities and impairments reflected in our study, with a high prevalence of as ADL dependency and dementia. This results in poor candidates for emergency surgery. Furthermore, lower physiological reserve may decrease their ability to cope with surgical stress and lead to more complications. Frailty scores are often used to assess surgical risk in the elderly population [14]. 30% of older people with frailty die within a year after having an emergency surgery compared to older patients who are not frail [12]. In our study that specifically evaluated perioperative outcomes following emergency colectomy, the mortality rate one year after surgery in patients ≥80 years who survived the operation was 22%; much higher than among younger patients.

This study identified several risk-factors for perioperative mortality that varied across age groups. In the below 60 age group, perioperative mortality was directly associated with the level of the severity of their condition in which surgery was performed, reflected by the ASA score and WBC count. In patients up to 79 years, ADL dependency became significant which is more common in this age group. In the oldest group (≥80 years) additional risk-factors were identified in univariate analysis, including dementia. However, did not reach statistical significance in multivariate analysis. This may be because these factors are generally more prevalent in patients older than 80 and that the combination of all these factors and not a specific single factor leads to a substantial increase in perioperative mortality in this age group.

Previous studies in elderly patients undergoing emergent and elective general surgery, trauma surgery, vascular surgery or orthopedic surgery for hip fractures, have reported higher mortality rates when dementia was present [15,16,17,18]. ASA score, another non-modifiable risk factor, has been linked to higher mortality after acute surgery in the elderly [19,20]. This factor could account for the multiple co-morbidities often seen in the elderly population.

To the best of our knowledge, this is the first study that specifically examined ADL dependency as a risk-factor for mortality after colorectal surgery. Other studies have shown frailty to be associated with mortality, but not specifically ADL dependence [21,22]. ADL dependence is a risk factor for 30-day mortality probably because these patients are less physically active, have worse preconditioning and are less likely to adhere to recovery activities such as early mobilization, and physical and respiratory therapy. This makes them potentially more prone to life-threatening complications such as venous thromboembolism, and pulmonary and urinary tract complications.

Several studies evaluated patients who underwent emergency surgery and identified sepsis as a risk-factor for 30 day-mortality [23,24]. These findings may imply it is important to appropriately resuscitate patients prior to surgery with timely antibiotics and fluids prior to surgery in septic patients. These recommendations are in line with the Surviving Sepsis campaign, which has outlined the importance of these interventions and should be generalized to include patients with underlying sepsis requiring emergency colectomies [25].

The limitations of this study include its retrospective nature and single academic center setting with general surgeons of varying levels of colorectal surgery training performing emergency colonic resections. A previous study on emergent colectomies showed lower rates of postoperative morbidity and mortality when performed by a trained colorectal surgeon [26]. Thus, these results may not apply to institutions where emergent colonic resections are performed by dedicated colorectal surgeons.

Furthermore, the retrospective nature of this study precluded calculation of objective scores such as a frailty score or Katz index of independence in ADL. Calculation of these scores requires data which is not routinely collected in the medical records of acutely admitted patients. Despite these limitations, this study confirms high mortality rates after emergent colonic resections in older patients and identifies potential the risk-factors that could be applicable to any practice performing emergency colectomies in the elderly. Larger scale and possibly multi-center studies may further elucidate mortality rates and other risk-factors for mortality in this growing population.

Our findings may encourage colorectal cancer screening in the elderly population, which can lead to early diagnosis and treatment with elective surgery and potentially lower mortality. Furthermore, physicians and healthcare workers caring for this population should be familiar with the signs and symptoms of large bowel pathologies that can lead to prompt diagnosis and timely transfer to emergency healthcare facilities.

Surgeons are increasingly consulted on elderly patients with acute surgical pathologies, often requiring surgery with large bowel resection. When considering the potential risks of surgery, the surgeon must consider the patient’s age, general status, comorbidities, and surgical problems, to name a few. This information may be valuable for the surgeon and critical surgical teams preparing a patient for acute care surgery and when discussing expectations regarding surgical outcomes with patients and their families.

## 5. Conclusions

Increasing life expectancy coupled with the high prevalence of colorectal pathologies have led to a dramatic rise in the number of elderly patients undergoing emergency colorectal surgery. Perioperative mortality increases gradually from the age of 60, reaching a high percentage in patients over 80 years old. In this age group, the mortality rate is extremely high and is believed to be associated with a combination of risk-factors related to the severity of their presentation and pre-existing conditions typical of the elderly population. Surgeons treating the older population should be aware of higher mortality risks and try to address modifiable risk-factors prior to undertaking emergent colorectal resections, when applicable. Screening of the elderly population for colonic pathologies can result in early diagnosis that would lead to elective surgeries with decreased mortality.

## Figures and Tables

**Table 1:** Total procedures performed.

Procedure	Total Cohort *n* = 613
Right colectomy, *n* (%)	178 (29)
Hartmann’s procedure, *n* (%)	178 (29)
Left colectomy, *n* (%)	71 (11.5)
Sub-total colectomy, *n* (%)	54 (9)
Cecectomy, *n* (%)	53 (8)
Sigmoidectomy, *n* (%)	29 (4.7)
Subtotal colectomy with ileostomy, *n* (%)	25 (4)
Total colectomy, *n* (%)	15 (2.4)
Anterior resection, *n* (%)	9 (1.4)
Abdominoperineal resection, *n* (%)	1 (0.1)

**Table 2:** Risk-factors for 30-day mortality according to age groups- univariate analysis.

	18–59			60–69			70–79			80+		
	Survive *n*= 150 (96.8%)	30d Death *n*= 5 (3.2%)	*p* Value	Survive *n*= 113 (89%)	30d Death *n*= 14 (11%)	*p* Value	Survive *n*= 87 (70.7%)	30d Death *n*= 36 (29.3%)	*p* Value	Survive *n*= 130 (62.2%)	30d Death *n*= 79 (37.8%)	*p* Value
Gender *n* (%)			1.000			0.392			0.877			0.107
Male	98 (65.3)	3 (60)		71 (62.8)	7 (50)		47 (54.0)	20 (55.6)		51 (39.2)	40 (50.6)	
Female	52 (34.7)	2 (40)		42 (37.2)	7 (50)		40 (46.0)	16 (44.4)		79 (60.8)	39 (49.4)	
BMI mean ± SD	--	--		28.62 ± 5.36	31.72 ± 6.98	0.162	27.23 ± 4.17	27.92 ± 7.53	0.766	25.49 ± 4.55	26.23 ± 6.32	0.787
ADL-dependent *n* (%)	10 (6.7)	1 (20)	0.311	**12 (10.6)**	**8 (57.1)**	**0.000**	**7 (8.0)**	**12 (33.3)**	**0.000**	**30 (23.1)**	**37 (47.4)**	**0.000**
Dementia *n* (%)	3 (2)	1 (20)	0.124	4 (3.5)	2 (14.3)	0.131	**3 (3.4)**	**8 (22.2)**	**0.002**	**21 (16.2)**	**29 (37.2)**	**0.001**
Cancer *n* (%)	121 (80.7)	4 (80)	1.000	73 (64.6)	12 (85.7)	0.141	58 (66.7)	24 (66.7)	1.000	**63 (48.5)**	**53 (67.1)**	**0.009**
ASA score *n* (%)			**0.008**			**0.000**			**0.032**			**0.001**
1	**68 (45.3)**	**1 (20)**		**19 (16.8)**	**0 (0)**		**6 (6.9)**	**2 (5.6)**		**8 (6.2)**	**5 (6.3)**	
2	**54 (36.0)**	**2 (40)**		**53 (46.9)**	**5 (35.7)**		**39 (44.8)**	**10 (27.8)**		**54 (41.5)**	**13 (16.5)**	
3	**21 (14)**	**0 (0)**		**36 (31.9)**	**4 (28.6)**		**36 (41.4)**	**15 (41.7)**		**64 (49.2)**	**50 (63.3)**	
4	**7 (4.7)**	**2 (40)**		**5 (4.4)**	**5 (35.7)**		**6 (6.9)**	**9 (25.0)**		**4 (3.1)**	**10 (12.7)**	
5	**0 (0)**	**0 (0)**		**0 (0)**	**0 (0)**		**0 (0)**	**0 (0)**		**0**	**1 (1.3)**	
Charlson score mean ± SD	1.26 ± 2.52	2.60 ± 3.209	0.182	2.16 ± 2.52	2.64 ± 2.24	0.173	2.77 ± 2.69	2.97 ± 2.75	0.650	3.10 ± 3.23	3.33 ± 2.79	0.128
LOS mean ± SD	13.81 ± 12.54	7.00 ± 5.38	0.087	17.23 ± 16.34	11.43 ± 8.76	0.195	**14.86 ± 12.30**	**7.25 ± 8.54**	**0.001**	**18.01 ± 16.43**	**9.41 ± 8.68**	**0.000**
Blood transfusion *n* (%)	16 (10.7)	2 (40)	0.103	16 (14.2)	4 (28.6)	0.234	12 (13.8)	8 (22.2)	0.249	25 (19.2)	16 (20.3)	0.857
Hemoglobin mean ± SD	13.08 ± 2.21	12.54 ± 2.87	0.594	13.05 ± 2.16	12.01 ± 1.98	0.101	12.21 ± 2.05	12.10 ± 2.54	0.818	11.81 ± 1.89	13.38 ± 12.43	0.385
WBC mean ± SD	**13.09 ± 5.46**	**19.15 ± 6.50**	**0.017**	12.32 ± 5.25	17.68 ± 16.65	0.270	12.75 ± 4.57	14.54 ± 8.04	0.247	**12.13 ± 5.27**	**14.83 ± 8.33**	**0.035**
Creatinine mean ± SD	1.19 ± 1.43	2.75 ± 3.50	0.441	1.21 ± 0.91	1.9 ± 1.71	0.217	1.306 ± 0.927	1.946 ± 1.56	0.058	**1.25 ± 0.52**	**1.83 ± 1.60**	**0.000**
Albumin mean ± SD	--	--		3.03 ± 0.83	2.45 ± 0.49	0.375	2.87 ± 0.55	2.83 ± 0.66	0.901	**2.95 ± 0.69**	**2.34 ± 0.72**	**0.017**
Surgery specific cause			0.084			0.259			0.074			0.286
Obstruction, *n* (%)	39 (29.9)	3 (60)		50 (44.2)	4 (28.6)		43 (51.8)	10 (30.3)		75 (58.1)	37 (48.1)	
Perforation, *n* (%)	65 (44.8)	1 (20)		40 (35.4)	5 (35.7)		28 (33.7)	18 (54.5)		37 (28.7)	22 (28.6)	
Ischemia, *n* (%)	3 (2.1)	1 (20)		9 (8.0)	4 (28.6)		5 (6.0)	5 (15.2)		11 (8.5)	11 (14.3)	
Infection, *n* (%)	16 (11.0)	0 (0)		9 (8.0)	1 (7.1)		4 (4.8)	0 (0)		5 (3.9)	4 (5.2)	
Inflammation, *n* (%)	21 (14.5)	0 (0)		3 (2.7)	0 (0)		1 (1.2)	0 (0)		0 (0)	0 (0)	
Bleeding, *n* (%)	1 (0.7)	0 (0)		2 (1.8)	0 (0)		2 (2.4)	0 (0)		1 (0.8)	3 (3.9)	

Statistical significant values are in bold.

**Table 3:** Multivariable regression analysis for risk-factors for 30-day mortality.

18–59				60–69				70–79				≥80			
Variables in the Model	Adjusted OR	95% CI	*p* Value	Variables in the Model	Adjusted OR	95% CI	*p* Value	Variables in the Model	Adjusted OR	95% CI	*p* Value	Variables in the Model	Adjusted OR	95% CI	*p* Value
**WBC**	**1.215**	**1.040–1.421**	**0.014**	**ASA**	**2.459**	**1.057–5.72**	**0.037**	**ASA**	**2.428**	**1.085–5.43**	**0.031**	ASA	1.503	0.139–16.29	0.738
**ASA**	**2.675**	**0.943–7.584**	**0.064**	**ADL**	**6.773**	**1.652–27.76**	**0.008**	**ADL**	**9.664**	**1.536–60.79**	**0.016**	ADL	2.030	0.119–34.49	0.624
Blood transfusion	2.156	0.219–21.194	0.510	Dementia	1.113	0.123- 10.08	0.924	Dementia	0.848	0.089–8.04	0.886	Dementia	3.988	0.150–105.78	0.408
Specific cause	0.258	0.052–1.270	0.096	Hemoglobin	0.841	0.612- 1.15	0.288	Creatinine	1.344	0.894–2.02	0.155	Cancer	2.370	0.083–67.309	0.613
Dementia	13.164	0.722–240.149	0.082									WBC	1.285	0.982–1.681	0.068
												Creatinine	4.169	0.617–28.16	0.143
												Albumin	0.050	0.002–1.112	0.058

CI, confidence interval; OR, odds ratio; WBC, white blood cell count; ASA, American Society of Anesthesiologists; ADL, activities of daily living. Statistically significant variables and values are in bold.

## Data Availability

The data presented in this study are available on request from the corresponding author.
